# Acetonitrile­dicarbon­yl(η^5^-penta­methyl­cyclo­penta­dien­yl)iron(II) tetra­fluorido­borate

**DOI:** 10.1107/S1600536811021350

**Published:** 2011-06-18

**Authors:** Cyprian M. M’thiruaine, Holger B. Friedrich, Evans O. Changamu, Muhammad D. Bala

**Affiliations:** a School of Chemistry, University of KwaZulu-Natal, Westville Campus, Private Bag X54001, Durban 4000, South Africa; b Chemistry Department, Kenyatta University, PO Box 43844, Nairobi, Kenya

## Abstract

In the structure of the title compound, [Fe{η^5^-C_5_(CH_3_)_5_}(NCCH_3_)(CO)_2_]BF_4_, the arrangement of ligands around the Fe atom is in a pseudo-octa­hedral three-legged piano-stool fashion in which the penta­methyl­cyclo­penta­dienyl (Cp*) ligand occupies three apical coordination sites, while the two carbonyl and one acetonitrile ligands form the basal axes of the coordination. The Fe—N bond length is 1.924 (3) Å and the Fe—Cp* centroid distance is 1.722 Å.

## Related literature

For the synthetic route to the title compound, see: Catheline & Astruc (1984 ▶). For the structures of related analogues based on the (η^5^-C_5_H_5_) moiety, see: Callan *et al.* (1987 ▶) for aceto­nitrile coordination *via* carbon; Fadel *et al.* (1979 ▶) for aceto­nitrile coordination *via* nitro­gen. For our previous work in this area, see: M’thiruaine, Friedrich, Changamu & Bala (2011 ▶); M’thiruaine, Friedrich, Changamu & Omondi (2011 ▶). 
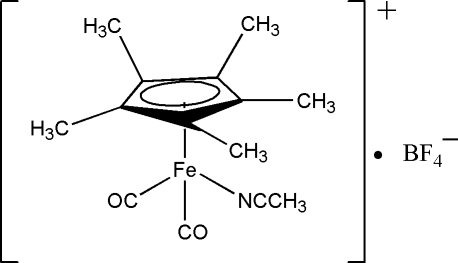


## Experimental

### 

#### Crystal data

[Fe(C_10_H_15_)(C_2_H_3_N)(CO)_2_]BF_4_
*M* *_r_* = 374.95
Orthorhombic, 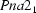

*a* = 17.6211 (17) Å
*b* = 6.5141 (7) Å
*c* = 14.5794 (13) Å
*V* = 1673.5 (3) Å^3^
*Z* = 4
Mo *K*α radiation
μ = 0.95 mm^−1^
*T* = 173 K
0.54 × 0.34 × 0.12 mm

#### Data collection

Bruker APEXII CCD diffractometer
Absorption correction: integration (*XPREP*; Bruker, 2005 ▶) *T*_min_ = 0.629, *T*_max_ = 0.895
9060 measured reflections
3496 independent reflections
2941 reflections with *I* > 2σ(*I*)
*R*_int_ = 0.044

#### Refinement

*R*[*F* ^2^ > 2σ(*F* ^2^)] = 0.044
*wR*(*F* ^2^) = 0.120
*S* = 1.08
3496 reflections
214 parameters
1 restraint
H-atom parameters constrained
Δρ_max_ = 0.76 e Å^−3^
Δρ_min_ = −0.39 e Å^−3^
Absolute structure: Flack (1983 ▶), 13942 Friedel pairs
Flack parameter: −0.02 (3)

### 

Data collection: *APEX2* (Bruker, 2005 ▶); cell refinement: *SAINT-Plus* (Bruker, 2005 ▶); data reduction: *SAINT-Plus*; program(s) used to solve structure: *SHELXTL* (Sheldrick, 2008 ▶); program(s) used to refine structure: *SHELXTL*; molecular graphics: *ORTEP-3* (Farrugia, 1997 ▶); software used to prepare material for publication: *SHELXTL*.

## Supplementary Material

Crystal structure: contains datablock(s) global, I. DOI: 10.1107/S1600536811021350/hg5040sup1.cif

Structure factors: contains datablock(s) I. DOI: 10.1107/S1600536811021350/hg5040Isup2.hkl

Additional supplementary materials:  crystallographic information; 3D view; checkCIF report

## Figures and Tables

**Fig. 1.**
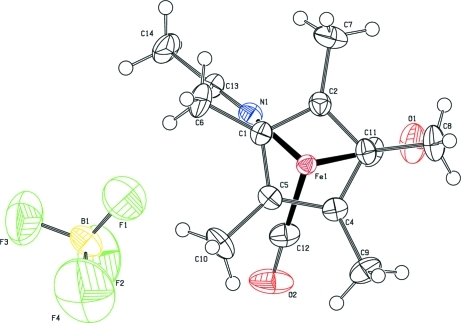
Molecular structure of the title compound with the atom numbering scheme. Displacement ellipsoids are drawn at the 50% probability level. H atoms are presented as small spheres of arbitrary radius.

## supplementary crystallographic information

### Comment

The title compound (I) was obtained as a side product in our ongoing investigation of the reactions of substitutionally unsaturated metal complexes with nitrogen donor ligands (M'thiruaine, Friedrich, Changamu & Bala, 2011; M'thiruaine, Friedrich, Changamu & Omondi, 2011). The compound has been previously reported as the product of oxidative cleavage of the Fe—Fe bond in [η^5^-C_5_(CH_3_)_5_Fe(CO)_2_]_2_ in acetonitrile and also as a product of the reaction between [η^5^-C_5_(CH_3_)_5_Fe(CO)_2_(THF)]BF_4_ and acetonitrile (Catheline & Astruc 1984), but its crystal structure has not been reported. Compound (I) crystallizes in an orthorhombic *Pna2_1_* space group, with four discrete molecular cations and four counteranions in the unit cell. The arrangement of ligands around Fe is in a pseudo-octahedral 3-legged piano stool fashion in which the pentamethylcyclopentadienyl moiety occupies three coordination sites while the two carbonyl ligands and acetonitrile nitrogen complete the coordination. The Fe—N bond length of 1.924 (3) Å, is close to the 1.91 (1)Å reported for [η^5^-C_5_H_5_Fe(CO)_2_(NCCH_3_)]BF_4_ (Fadel *et al.* 1979) but shorter than Fe—N bonds found in the pyrrol complex, [η^5^-C_5_H_5_Fe(CO)_2_(C_4_H_4_N)] (1.962 (3) Å), and in the aminoalkane complexes, [η^5^-C_5_H_5_Fe(CO)_2_(NH_2_(CH_2_)_n_CH_3_)]BF_4_ (n=2,3) (2.017 (8), 2.013 (3) and 2.006 (2) Å) (M'thiruaine, Friedrich, Changamu & Bala, 2011) and [{η^5^-C_5_H_5_Fe(CO)_2_}_2_(µ-(NH_2_CH_2_CH_2_NH_2_)](BF_4_)_2_ (2.0134 (17) and 2.0085 (18) Å) (M'thiruaine, Friedrich, Changamu & Omondi, 2011). It is also interesting to note that both Fe—C (Callan *et al.* 1987) and Fe—N (Fadel *et al.* 1979) coordination of the acetonitrile (NCCH_3_) molecule to Fe has been reported for Cp based complexes.

### Experimental

The title compound (I) was synthesized following the method of Catheline & Astruc (1984). Compound (I) was obtained as a yellow microcrystalline solid in an isolated yield of 92%.

Anal. Calc. for C_14_H_18_BF_4_FeNO_2_: C, 44.80; H, 4.80; N, 3.73. Found: C, 44.95; H, 4.13; N, 3.76%.

^1^H-NMR (400 MHz, CDCl_3_): δ 2.48 (s, 3H, NCCH_3_), 1.85 (s, 15H, C_5_(CH_3_)_5_).

^13^C-NMR (400 MHz, CDCl_3_): δ 4.68 (NCCH_3_) 9.54 (C_5_(CH_3_)_5_), 99.19 (C_5_(CH_3_)_5_), 210.08 (CO).

IR (solid state, cm^-1^): ν(CO) 2044; 1992, v(CN) 2299.

Melting point = 158–160 °C.

### Refinement

All H-atoms were refined using a riding model, with C—H = 0.98 Å and *U*_iso_(H) = 1.5*U*_eq_(C) for CH_3_.

### Crystal data

**Table:**

[Fe(C_10_H_15_)(C_2_H_3_N)(CO)_2_]BF_4_	*F*(000) = 768
*M**_r_* = 374.95	*D*_x_ = 1.488 Mg m^−^^3^
Orthorhombic, *P**n**a*2_1_	Mo *K*α radiation, λ = 0.71073 Å
Hall symbol: P 2c -2n	Cell parameters from 3473 reflections
*a* = 17.6211 (17) Å	θ = 2.3–28.2°
*b* = 6.5141 (7) Å	µ = 0.95 mm^−^^1^
*c* = 14.5794 (13) Å	*T* = 173 K
*V* = 1673.5 (3) Å^3^	Plate, yellow
*Z* = 4	0.54 × 0.34 × 0.12 mm

### Data collection

**Table:**

Bruker APEXII CCD diffractometer	3496 independent reflections
Radiation source: fine-focus sealed tube	2941 reflections with *I* > 2σ(*I*)
graphite	*R*_int_ = 0.044
φ and ω scans	θ_max_ = 28.0°, θ_min_ = 2.3°
Absorption correction: integration (*XPREP*; Bruker, 2005)	*h* = −23→22
*T*_min_ = 0.629, *T*_max_ = 0.895	*k* = −8→6
9060 measured reflections	*l* = −19→12

### Refinement

**Table:**

Refinement on *F*^2^	Secondary atom site location: difference Fourier map
Least-squares matrix: full	Hydrogen site location: inferred from neighbouring sites
*R*[*F*^2^ > 2σ(*F*^2^)] = 0.044	H-atom parameters constrained
*wR*(*F*^2^) = 0.120	*w* = 1/[σ^2^(*F*_o_^2^) + (0.0515*P*)^2^ + 1.372*P*] where *P* = (*F*_o_^2^ + 2*F*_c_^2^)/3
*S* = 1.08	(Δ/σ)_max_ = 0.001
3496 reflections	Δρ_max_ = 0.76 e Å^−^^3^
214 parameters	Δρ_min_ = −0.39 e Å^−^^3^
1 restraint	Absolute structure: Flack (1983), 13942 Friedel pairs
Primary atom site location: structure-invariant direct methods	Flack parameter: −0.02 (3)

### Special details

**Table:**

Experimental. Face indexed absorption corrections carried out with XPREP (Bruker 2005).
Geometry. All e.s.d.'s (except the e.s.d. in the dihedral angle between two l.s. planes) are estimated using the full covariance matrix. The cell e.s.d.'s are taken into account individually in the estimation of e.s.d.'s in distances, angles and torsion angles; correlations between e.s.d.'s in cell parameters are only used when they are defined by crystal symmetry. An approximate (isotropic) treatment of cell e.s.d.'s is used for estimating e.s.d.'s involving l.s. planes.
Refinement. Refinement of *F*^2^ against ALL reflections. The weighted *R*-factor *wR* and goodness of fit *S* are based on *F*^2^, conventional *R*-factors *R* are based on *F*, with *F* set to zero for negative *F*^2^. The threshold expression of *F*^2^ > σ(*F*^2^) is used only for calculating *R*-factors(gt) *etc*. and is not relevant to the choice of reflections for refinement. *R*-factors based on *F*^2^ are statistically about twice as large as those based on *F*, and *R*- factors based on ALL data will be even larger.

### Fractional atomic coordinates and isotropic or equivalent isotropic displacement parameters (Å^2^)

**Table:**

	*x*	*y*	*z*	*U*_iso_*/*U*_eq_	
C1	0.09216 (16)	0.7960 (5)	0.3098 (3)	0.0239 (6)	
C2	0.02658 (19)	0.8041 (6)	0.3681 (3)	0.0262 (8)	
C3	−0.03861 (16)	0.7629 (5)	0.3117 (4)	0.0257 (6)	
C4	−0.0122 (2)	0.7201 (6)	0.2209 (3)	0.0260 (8)	
C5	0.0690 (2)	0.7432 (6)	0.2201 (3)	0.0254 (7)	
C6	0.1726 (2)	0.8390 (7)	0.3396 (3)	0.0400 (11)	
H6A	0.2067	0.7375	0.3121	0.060*	
H6B	0.1759	0.8306	0.4066	0.060*	
H6C	0.1873	0.9769	0.3195	0.060*	
C7	0.0265 (3)	0.8621 (8)	0.4661 (3)	0.0427 (11)	
H7A	0.0329	1.0111	0.4716	0.064*	
H7B	0.0684	0.7925	0.4975	0.064*	
H7C	−0.0217	0.8214	0.4940	0.064*	
C8	−0.1200 (2)	0.7710 (7)	0.3400 (3)	0.0426 (12)	
H8A	−0.1402	0.9086	0.3282	0.064*	
H8B	−0.1241	0.7400	0.4056	0.064*	
H8C	−0.1491	0.6698	0.3048	0.064*	
C9	−0.0613 (3)	0.6839 (7)	0.1391 (3)	0.0379 (10)	
H9A	−0.0798	0.8157	0.1156	0.057*	
H9B	−0.1046	0.5978	0.1566	0.057*	
H9C	−0.0318	0.6143	0.0913	0.057*	
C10	0.1205 (3)	0.7291 (7)	0.1393 (3)	0.0405 (11)	
H10A	0.1273	0.8659	0.1126	0.061*	
H10B	0.0982	0.6371	0.0934	0.061*	
H10C	0.1698	0.6750	0.1588	0.061*	
C11	−0.0393 (2)	0.3667 (7)	0.3676 (3)	0.0331 (9)	
C12	0.0500 (2)	0.3192 (6)	0.2248 (3)	0.0335 (9)	
C13	0.1669 (2)	0.3605 (6)	0.4320 (3)	0.0284 (8)	
C14	0.2331 (2)	0.3052 (8)	0.4861 (4)	0.0452 (12)	
H14A	0.2780	0.3025	0.4465	0.068*	
H14B	0.2255	0.1692	0.5133	0.068*	
H14C	0.2404	0.4065	0.5350	0.068*	
B1	0.2910 (3)	0.2863 (8)	0.1717 (4)	0.0432 (13)	
N1	0.11513 (17)	0.4097 (5)	0.3903 (2)	0.0265 (7)	
O1	−0.08715 (17)	0.2810 (5)	0.4027 (3)	0.0530 (9)	
O2	0.0583 (3)	0.2069 (5)	0.1657 (3)	0.0575 (10)	
F1	0.2517 (3)	0.3976 (10)	0.2318 (4)	0.126 (2)	
F2	0.2748 (3)	0.0894 (7)	0.1790 (5)	0.1188 (19)	
F3	0.3707 (2)	0.3066 (8)	0.1896 (4)	0.1167 (19)	
F4	0.2819 (4)	0.3427 (15)	0.0873 (5)	0.189 (4)	
Fe1	0.03492 (2)	0.50825 (7)	0.31236 (7)	0.02001 (13)	

### Atomic displacement parameters (Å^2^)

**Table:**

	*U*^11^	*U*^22^	*U*^33^	*U*^12^	*U*^13^	*U*^23^
C1	0.0235 (13)	0.0212 (14)	0.0270 (16)	−0.0015 (11)	−0.0006 (18)	0.002 (2)
C2	0.0246 (16)	0.0246 (18)	0.029 (2)	0.0019 (14)	0.0012 (14)	−0.0014 (16)
C3	0.0218 (13)	0.0250 (14)	0.0302 (17)	0.0031 (11)	0.0037 (17)	0.004 (2)
C4	0.0314 (17)	0.0213 (18)	0.0254 (19)	0.0015 (14)	−0.0026 (15)	0.0044 (15)
C5	0.0299 (17)	0.0209 (17)	0.0255 (19)	0.0003 (14)	0.0035 (15)	0.0024 (15)
C6	0.0243 (16)	0.034 (2)	0.061 (3)	−0.0084 (16)	−0.0037 (16)	0.001 (2)
C7	0.058 (3)	0.043 (3)	0.026 (2)	0.009 (2)	0.0010 (19)	−0.007 (2)
C8	0.0250 (17)	0.044 (2)	0.059 (3)	0.0083 (16)	0.0072 (17)	0.008 (2)
C9	0.045 (2)	0.035 (2)	0.034 (2)	−0.0034 (19)	−0.0151 (19)	0.0082 (19)
C10	0.046 (2)	0.039 (2)	0.037 (2)	0.0038 (19)	0.0203 (19)	0.007 (2)
C11	0.0275 (17)	0.030 (2)	0.042 (3)	−0.0028 (15)	−0.0055 (16)	0.0076 (19)
C12	0.044 (2)	0.028 (2)	0.028 (2)	0.0049 (17)	−0.0081 (17)	0.0039 (18)
C13	0.0223 (16)	0.033 (2)	0.030 (2)	0.0013 (15)	−0.0033 (14)	0.0001 (17)
C14	0.032 (2)	0.055 (3)	0.049 (3)	0.015 (2)	−0.0176 (19)	−0.002 (2)
B1	0.056 (3)	0.032 (3)	0.042 (3)	0.009 (2)	0.013 (2)	0.002 (2)
N1	0.0285 (15)	0.0268 (16)	0.0242 (17)	0.0001 (13)	0.0015 (12)	0.0015 (13)
O1	0.0375 (16)	0.052 (2)	0.069 (2)	−0.0149 (15)	0.0089 (17)	0.0209 (19)
O2	0.096 (3)	0.0380 (19)	0.038 (2)	0.0189 (19)	−0.0112 (19)	−0.0144 (17)
F1	0.094 (3)	0.146 (4)	0.137 (5)	0.048 (3)	−0.003 (3)	−0.081 (4)
F2	0.100 (3)	0.068 (3)	0.188 (6)	−0.008 (3)	0.037 (3)	0.015 (4)
F3	0.067 (2)	0.107 (4)	0.175 (5)	0.009 (2)	0.000 (3)	−0.041 (4)
F4	0.192 (7)	0.256 (9)	0.120 (6)	0.030 (6)	0.020 (5)	0.109 (6)
Fe1	0.01998 (19)	0.0212 (2)	0.0188 (2)	−0.00051 (17)	−0.0013 (3)	0.0014 (2)

### Geometric parameters (Å, °)

**Table:**

C1—C5	1.413 (6)	C8—H8B	0.9800
C1—C2	1.436 (5)	C8—H8C	0.9800
C1—C6	1.508 (5)	C9—H9A	0.9800
C1—Fe1	2.129 (3)	C9—H9B	0.9800
C2—C3	1.438 (5)	C9—H9C	0.9800
C2—C7	1.477 (6)	C10—H10A	0.9800
C2—Fe1	2.097 (4)	C10—H10B	0.9800
C3—C4	1.431 (7)	C10—H10C	0.9800
C3—C8	1.493 (5)	C11—O1	1.134 (5)
C3—Fe1	2.105 (3)	C11—Fe1	1.791 (4)
C4—C5	1.439 (5)	C12—O2	1.140 (6)
C4—C9	1.493 (6)	C12—Fe1	1.793 (5)
C4—Fe1	2.091 (4)	C13—N1	1.143 (5)
C5—C10	1.490 (5)	C13—C14	1.453 (5)
C5—Fe1	2.124 (4)	C14—H14A	0.9800
C6—H6A	0.9800	C14—H14B	0.9800
C6—H6B	0.9800	C14—H14C	0.9800
C6—H6C	0.9800	B1—F4	1.295 (9)
C7—H7A	0.9800	B1—F2	1.318 (7)
C7—H7B	0.9800	B1—F1	1.332 (7)
C7—H7C	0.9800	B1—F3	1.433 (7)
C8—H8A	0.9800	N1—Fe1	1.924 (3)
			
C5—C1—C2	108.9 (3)	H9A—C9—H9C	109.5
C5—C1—C6	125.7 (4)	H9B—C9—H9C	109.5
C2—C1—C6	125.4 (4)	C5—C10—H10A	109.5
C5—C1—Fe1	70.4 (2)	C5—C10—H10B	109.5
C2—C1—Fe1	68.9 (2)	H10A—C10—H10B	109.5
C6—C1—Fe1	127.1 (3)	C5—C10—H10C	109.5
C1—C2—C3	107.3 (4)	H10A—C10—H10C	109.5
C1—C2—C7	125.6 (4)	H10B—C10—H10C	109.5
C3—C2—C7	126.9 (4)	O1—C11—Fe1	178.5 (4)
C1—C2—Fe1	71.4 (2)	O2—C12—Fe1	176.2 (4)
C3—C2—Fe1	70.3 (2)	N1—C13—C14	178.0 (5)
C7—C2—Fe1	127.6 (3)	C13—C14—H14A	109.5
C4—C3—C2	107.8 (3)	C13—C14—H14B	109.5
C4—C3—C8	125.1 (4)	H14A—C14—H14B	109.5
C2—C3—C8	127.0 (5)	C13—C14—H14C	109.5
C4—C3—Fe1	69.5 (2)	H14A—C14—H14C	109.5
C2—C3—Fe1	69.69 (19)	H14B—C14—H14C	109.5
C8—C3—Fe1	128.2 (3)	F4—B1—F2	109.0 (7)
C3—C4—C5	108.1 (3)	F4—B1—F1	113.9 (6)
C3—C4—C9	125.6 (4)	F2—B1—F1	111.4 (6)
C5—C4—C9	125.9 (4)	F4—B1—F3	105.6 (6)
C3—C4—Fe1	70.6 (2)	F2—B1—F3	106.7 (5)
C5—C4—Fe1	71.3 (2)	F1—B1—F3	109.9 (5)
C9—C4—Fe1	129.5 (3)	C13—N1—Fe1	174.2 (3)
C1—C5—C4	107.8 (3)	C11—Fe1—C12	94.3 (2)
C1—C5—C10	124.8 (4)	C11—Fe1—N1	95.67 (17)
C4—C5—C10	127.3 (4)	C12—Fe1—N1	94.73 (16)
C1—C5—Fe1	70.8 (2)	C11—Fe1—C4	109.68 (17)
C4—C5—Fe1	68.8 (2)	C12—Fe1—C4	93.34 (18)
C10—C5—Fe1	128.9 (3)	N1—Fe1—C4	152.70 (14)
C1—C6—H6A	109.5	C11—Fe1—C2	104.35 (18)
C1—C6—H6B	109.5	C12—Fe1—C2	156.57 (19)
H6A—C6—H6B	109.5	N1—Fe1—C2	97.42 (15)
C1—C6—H6C	109.5	C4—Fe1—C2	67.22 (16)
H6A—C6—H6C	109.5	C11—Fe1—C3	87.62 (17)
H6B—C6—H6C	109.5	C12—Fe1—C3	129.0 (2)
C2—C7—H7A	109.5	N1—Fe1—C3	135.88 (18)
C2—C7—H7B	109.5	C4—Fe1—C3	39.87 (19)
H7A—C7—H7B	109.5	C2—Fe1—C3	40.03 (15)
C2—C7—H7C	109.5	C11—Fe1—C5	149.54 (17)
H7A—C7—H7C	109.5	C12—Fe1—C5	90.14 (18)
H7B—C7—H7C	109.5	N1—Fe1—C5	114.01 (14)
C3—C8—H8A	109.5	C4—Fe1—C5	39.90 (15)
C3—C8—H8B	109.5	C2—Fe1—C5	66.62 (15)
H8A—C8—H8B	109.5	C3—Fe1—C5	66.63 (16)
C3—C8—H8C	109.5	C11—Fe1—C1	143.80 (19)
H8A—C8—H8C	109.5	C12—Fe1—C1	121.48 (19)
H8B—C8—H8C	109.5	N1—Fe1—C1	87.48 (14)
C4—C9—H9A	109.5	C4—Fe1—C1	66.16 (15)
C4—C9—H9B	109.5	C2—Fe1—C1	39.71 (14)
H9A—C9—H9B	109.5	C3—Fe1—C1	66.28 (12)
C4—C9—H9C	109.5	C5—Fe1—C1	38.80 (17)
			
C5—C1—C2—C3	−2.2 (4)	C1—C2—Fe1—C4	−79.6 (2)
C6—C1—C2—C3	177.2 (3)	C3—C2—Fe1—C4	37.4 (2)
Fe1—C1—C2—C3	−61.4 (2)	C7—C2—Fe1—C4	159.4 (4)
C5—C1—C2—C7	−177.4 (4)	C1—C2—Fe1—C3	−117.0 (4)
C6—C1—C2—C7	2.1 (6)	C7—C2—Fe1—C3	121.9 (5)
Fe1—C1—C2—C7	123.4 (4)	C1—C2—Fe1—C5	−36.1 (2)
C5—C1—C2—Fe1	59.2 (2)	C3—C2—Fe1—C5	81.0 (3)
C6—C1—C2—Fe1	−121.3 (4)	C7—C2—Fe1—C5	−157.1 (4)
C1—C2—C3—C4	2.8 (4)	C3—C2—Fe1—C1	117.0 (4)
C7—C2—C3—C4	177.9 (4)	C7—C2—Fe1—C1	−121.0 (4)
Fe1—C2—C3—C4	−59.3 (2)	C4—C3—Fe1—C11	−125.1 (3)
C1—C2—C3—C8	−174.8 (3)	C2—C3—Fe1—C11	115.8 (3)
C7—C2—C3—C8	0.3 (7)	C8—C3—Fe1—C11	−5.9 (5)
Fe1—C2—C3—C8	123.1 (4)	C4—C3—Fe1—C12	−31.6 (3)
C1—C2—C3—Fe1	62.1 (3)	C2—C3—Fe1—C12	−150.7 (3)
C7—C2—C3—Fe1	−122.8 (4)	C8—C3—Fe1—C12	87.6 (5)
C2—C3—C4—C5	−2.4 (4)	C4—C3—Fe1—N1	139.2 (2)
C8—C3—C4—C5	175.3 (3)	C2—C3—Fe1—N1	20.1 (3)
Fe1—C3—C4—C5	−61.8 (2)	C8—C3—Fe1—N1	−101.6 (4)
C2—C3—C4—C9	−175.4 (4)	C2—C3—Fe1—C4	−119.1 (3)
C8—C3—C4—C9	2.3 (6)	C8—C3—Fe1—C4	119.2 (5)
Fe1—C3—C4—C9	125.2 (4)	C4—C3—Fe1—C2	119.1 (3)
C2—C3—C4—Fe1	59.4 (2)	C8—C3—Fe1—C2	−121.7 (6)
C8—C3—C4—Fe1	−122.9 (4)	C4—C3—Fe1—C5	38.2 (2)
C2—C1—C5—C4	0.8 (4)	C2—C3—Fe1—C5	−80.9 (2)
C6—C1—C5—C4	−178.7 (3)	C8—C3—Fe1—C5	157.3 (5)
Fe1—C1—C5—C4	59.1 (2)	C4—C3—Fe1—C1	80.6 (2)
C2—C1—C5—C10	177.1 (4)	C2—C3—Fe1—C1	−38.4 (2)
C6—C1—C5—C10	−2.4 (6)	C8—C3—Fe1—C1	−160.2 (5)
Fe1—C1—C5—C10	−124.6 (4)	C1—C5—Fe1—C11	115.2 (4)
C2—C1—C5—Fe1	−58.3 (3)	C4—C5—Fe1—C11	−3.6 (5)
C6—C1—C5—Fe1	122.2 (3)	C10—C5—Fe1—C11	−125.1 (5)
C3—C4—C5—C1	1.0 (4)	C1—C5—Fe1—C12	−146.1 (2)
C9—C4—C5—C1	174.0 (4)	C4—C5—Fe1—C12	95.0 (2)
Fe1—C4—C5—C1	−60.3 (3)	C10—C5—Fe1—C12	−26.4 (4)
C3—C4—C5—C10	−175.2 (4)	C1—C5—Fe1—N1	−50.9 (2)
C9—C4—C5—C10	−2.2 (7)	C4—C5—Fe1—N1	−169.7 (2)
Fe1—C4—C5—C10	123.5 (4)	C10—C5—Fe1—N1	68.8 (4)
C3—C4—C5—Fe1	61.3 (2)	C1—C5—Fe1—C4	118.8 (3)
C9—C4—C5—Fe1	−125.7 (4)	C10—C5—Fe1—C4	−121.5 (5)
C3—C4—Fe1—C11	60.2 (3)	C1—C5—Fe1—C2	36.89 (19)
C5—C4—Fe1—C11	178.1 (2)	C4—C5—Fe1—C2	−81.9 (2)
C9—C4—Fe1—C11	−60.4 (4)	C10—C5—Fe1—C2	156.6 (4)
C3—C4—Fe1—C12	155.9 (2)	C1—C5—Fe1—C3	80.7 (2)
C5—C4—Fe1—C12	−86.2 (3)	C4—C5—Fe1—C3	−38.1 (2)
C9—C4—Fe1—C12	35.3 (4)	C10—C5—Fe1—C3	−159.6 (4)
C3—C4—Fe1—N1	−97.0 (4)	C4—C5—Fe1—C1	−118.8 (3)
C5—C4—Fe1—N1	20.8 (4)	C10—C5—Fe1—C1	119.7 (5)
C9—C4—Fe1—N1	142.4 (4)	C5—C1—Fe1—C11	−129.0 (3)
C3—C4—Fe1—C2	−37.6 (2)	C2—C1—Fe1—C11	−8.6 (4)
C5—C4—Fe1—C2	80.3 (2)	C6—C1—Fe1—C11	110.5 (4)
C9—C4—Fe1—C2	−158.2 (4)	C5—C1—Fe1—C12	40.8 (3)
C5—C4—Fe1—C3	117.9 (3)	C2—C1—Fe1—C12	161.2 (2)
C9—C4—Fe1—C3	−120.6 (5)	C6—C1—Fe1—C12	−79.7 (5)
C3—C4—Fe1—C5	−117.9 (3)	C5—C1—Fe1—N1	134.8 (2)
C9—C4—Fe1—C5	121.6 (5)	C2—C1—Fe1—N1	−104.8 (2)
C3—C4—Fe1—C1	−81.0 (2)	C6—C1—Fe1—N1	14.3 (4)
C5—C4—Fe1—C1	36.9 (2)	C5—C1—Fe1—C4	−37.9 (2)
C9—C4—Fe1—C1	158.4 (4)	C2—C1—Fe1—C4	82.5 (2)
C1—C2—Fe1—C11	174.8 (2)	C6—C1—Fe1—C4	−158.4 (5)
C3—C2—Fe1—C11	−68.2 (3)	C5—C1—Fe1—C2	−120.4 (3)
C7—C2—Fe1—C11	53.7 (4)	C6—C1—Fe1—C2	119.1 (5)
C1—C2—Fe1—C12	−43.7 (5)	C5—C1—Fe1—C3	−81.7 (3)
C3—C2—Fe1—C12	73.3 (5)	C2—C1—Fe1—C3	38.7 (3)
C7—C2—Fe1—C12	−164.7 (4)	C6—C1—Fe1—C3	157.9 (5)
C1—C2—Fe1—N1	76.9 (2)	C2—C1—Fe1—C5	120.4 (3)
C3—C2—Fe1—N1	−166.0 (2)	C6—C1—Fe1—C5	−120.5 (5)
C7—C2—Fe1—N1	−44.1 (4)		
